# Nephrogenic Systemic Fibrosis as a Complication after Gadolinium-Containing Contrast Agents: A Rapid Review

**DOI:** 10.3390/ijerph18063000

**Published:** 2021-03-15

**Authors:** Sandra Lange, Wioletta Mędrzycka-Dąbrowska, Katarzyna Zorena, Sebastian Dąbrowski, Daniel Ślęzak, Anna Malecka-Dubiela, Przemysław Rutkowski

**Affiliations:** 1 Department of Anesthesiology and Intensive Care, Hospitals Tczewskie SA, 30 Stycznia 57, 83-110 Tczew, Poland; langa94@gumed.edu.pl; 2 Department of Anaesthesiology Nursing and Intensive Care, Faculty of Health Sciences, Medical University in Gdansk, Dębinki 7, 80-211 Gdańsk, Poland; 3 Department of Immunobiology and Environment Microbiology, Faculty of Health Sciences, Medical University of Gdańsk, Dębinki 7, 80-211 Gdańsk, Poland; katarzyna.zorena@gumed.edu.pl; 4 Department of Medical Rescue, Faculty of Health Sciences, Medical University of Gdańsk, Dębinki 7, 80-211 Gdańsk, Poland; sebastian.dabrowski@gumed.edu.pl (S.D.); daniel.slezak@gumed.edu.pl (D.Ś.); 5 Department of Internal and Pediatric Nursing, Faculty of Health Sciences, Medical University of Gdańsk, Dębinki 7, 80-211 Gdańsk, Poland; anna.malecka-dubiela@gumed.edu.pl (A.M.-D.); przemyslaw.rutkowski@gumed.edu.pl (P.R.)

**Keywords:** nephrogenic systemic fibrosis, gadolinium, renal failure

## Abstract

**Introduction**: Nephrogenic systemic fibrosis (NFS) is a generalized disorder occurring in people with kidney failure. This new disease entity can lead to significant disability or even death. Gadolinium-associated systemic fibrosis is related to exposure to contrast agents used for magnetic resonance imaging. The aim of this study was to review the literature in available scientific databases on NFS—complication after gadolinium-containing contrast agents. **Methods**: PubMed and Cochrane Library databases were searched using adequate key words. A literature review of the described cases of NSF occurrence after exposure to gadolinium-containing contrast agents was performed. A review followed the Preferred Reporting Items for Systematic Reviews and Meta-Analyses (PRISMA) statement. A review written protocol was not drafted. **Results**: Originally, 647 studies were searched in scientific databases. After rejecting the duplicate results, 515 results were obtained. Finally, nine studies were included in the review. A total of 173 cases with NSF were included in the analysis. The majority of patients were undergoing dialysis. The contrast agent used for MRI was most often gadodiamide and gadopentetate dimeglumine. The time from exposure to NSF symptoms was from two days to three years. Three authors pointed out other factors in their papers that could potentially influence the occurrence of NSF. These included: metabolic acidosis, ongoing infection, higher doses of erythropoietin and higher serum concentrations of ionized calcium and phosphate. Since 2008, the number of reported cases of NSF has decreased significantly. More recent guidelines and reports indicate that not all contrast agents are associated with the same risk of developing NSF. **Conclusions**: Most NSF occurs after exposure to linear contrast agents. Therefore, it is recommended to limit their use, especially in dialyzed patients and patients with a GFR < 30 mL/min.

## 1. Introduction

Nephrogenic systemic fibrosis (NFS), originally called nephrogenic fibrosing dermopathy (NFD), is an entity discovered in 1997 and described in 2000 in The Lancet [1]. NSF is a scleroderma-like fibrosing disorder that develops in the setting of renal insufficiency. The disorder was initially called nephrogenic fibrosing dermopathy, indicating the association with renal disease and the apparent involvement of the skin. Subsequently, it was found that the fibrosing process was present within muscles, myocardium, lungs and kidneys [2,3,4]. The course of the disease is mostly progressive. It may be accompanied by pain, muscle weakness, joint spasms that lead to cachexia, severe disability and, consequently, death [3,5]. The diagnosis of NSF is based on history, physical examination and differential diagnosis with many other conditions, e.g., systemic scleroderma, the Spanish toxic oil syndrome, amyloidosis or melorheostosis. Due to the low specificity of the histopathological picture, biopsy can serve as an auxiliary diagnosis for NSF [6]. The examined material shows the presence of thickened collagen fibers, between which there are gaps and thin bundles of collagen, accumulation of dendritic cells, proliferation of fibroblasts and elastic fibers [5,6,7]. Late in the disease, calcifications are found in the tissue [8,9]. The first to hypothesize that gadolinium-based contrast agents are involved in the pathogenesis of NSF was Grobner [5].

### 1.1. Contrast Agents Used for Magnetic Resonance Imaging

The most commonly used magnetic contrast agents for magnetic resonance imaging (MRI) are gadolinium chelates [10,11]. Their excretion from the body takes place through the kidneys. The half-life of a contrast agent in the body of a healthy person is 90 min. In patients with kidney failure, this period can be extended up to more than 30 h. The prolonged exposure time results in the dissociation of ions from the paramagnetic particles and their accumulation as deposits in the lymph nodes, bones, brain and liver. In addition, the presence of Gd3+ causes the activation of dendritic cells. These cells, producing transforming growth factor beta 1, initiate fibrosis and mobilize other dendritic cells. This may lead to an intensification of the fibrosis process [12]. Another mechanism that may initiate fibrosis is related to the phagocytosis of Gd3+ by macrophages. By releasing proinflammatory cytokines, these cells attract circulating fibrocytes, which then transform in the dermis into fibroblasts and initiate fibrosis [4,13]. An additional causative factor, especially in patients with renal failure, may be the transmetalation process. This process releases gadolinium by replacing Gd3+ in chelate molecules with systemic cations such as iron, zinc and copper [9,14]. The process of the release of free Gd3+ ions varies depending on the structure of the compound, which is part of the contrast agents [15,16]. There are two structural forms of chelates: linear and cyclic. A higher probability of the release of Gd3+ ions from chelate compounds is with linear contrast media, as opposed to cyclic chelate compounds, in which gadolinium is trapped inside. Since 2006, data have been collected on the basis of studies that confirm the relationship between NSF occurrence after exposure to contrast mediums containing gadodiamide and gadopentetate dimeglumine [8,11,17]. Cases of NFS after gadoversetamide application were also reported in the United States [10]. Due to the high risk of NSF development after exposure to these two contrast agents, their use has become a contraindication in patients with stage 4 and 5 chronic kidney disease (CKD). They should be used with particular caution in patients with CKD 3 [18]. The Committee for Medicinal Products for Human Use (CHMP) and American College of Radiology Classification (ACR) has divided the available contrast media on the public market according to NSF risk [19]. These agents are described in Table 1.

### 1.2. Aim

The aim of the study was to review the literature in available scientific databases on NFS—complication after gadolinium-containing contrast agents.

## 2. Methods

### 2.1. Study Design

A rapid review was carried out from July to December 2020.

### 2.2. Definition of a Rapid Review

A formal definition for a rapid review does not exist. As such, we used the following working definition: “a rapid review is a type of knowledge synthesis in which components of the systematic review process are simplified or omitted to produce information in a short period of time” [20].

### 2.3. Search Strategy

Searches were performed by two expert health science informationists. The “PICO” format was applied, as follows: (a) patients (patients undergoing gadolinium-containing contrast agent enhanced—MRI), (b) intervention (gadolinium-containing contrast agent enhanced—MRI), (c) comparison (not applicable) and (d) outcome (NSF). The following words were used for searching verification: nephrogenic systemic fibrosis, gadolinium, renal failure. Single keywords were introduced, as well as their combination with AND, OR and both operators (Table 2). The number of articles obtained during each search test was limited to studies conducted between 2006 and 2020. Strict inclusion and exclusion criteria were applied. The last search was conducted on 30 December 2020. Eventually, 9 articles were included in our review, which included two identical papers. Searches were performed by two expert health science informationists. Discrepancies were resolved through discussion.

### 2.4. Study Selection

Inclusion criteria: articles describing studies conducted in patients with kidney disease who have undergone intravenous MRI with a gadolinium-based contrast agent and who have developed NSF. Exclusion criteria: animal studies, articles published in languages other than English and articles to which the full version could not be accessed.

### 2.5. Screening Process

The quality of articles selected for review was assessed using the Newcastle–Ottawa Scale (NOS) [21]. The articles that were reviewed received 6–8 points (Table 3).

We applied the AMSTAR 2 quality appraisal checklist for systematic reviews and the Preferred Reporting Items for Systematic Reviews and Meta-Analyses (PRISMA) [28]. As this review does not include meta-analyses, any related AMSTAR 2 or PRISMA checklist items were considered inapplicable. A summary of the methodological quality assessment using the AMSTAR 2 checklist is presented in Table 4. The contents of two electronic databases, PubMed and Cochrane Library, were searched.

### 2.6. Data Extraction

The study was evaluated using a formalized form of data collection, which included the following data: first author, year of publication, number of NSF cases, average age of subjects, treatment of kidney disease, contrast agent used in the study and its amount, time of occurrence of NSF symptoms from exposure to contrast medium, as well as other potential factors that may cause NSF.

## 3. Results

**Figure 1:**
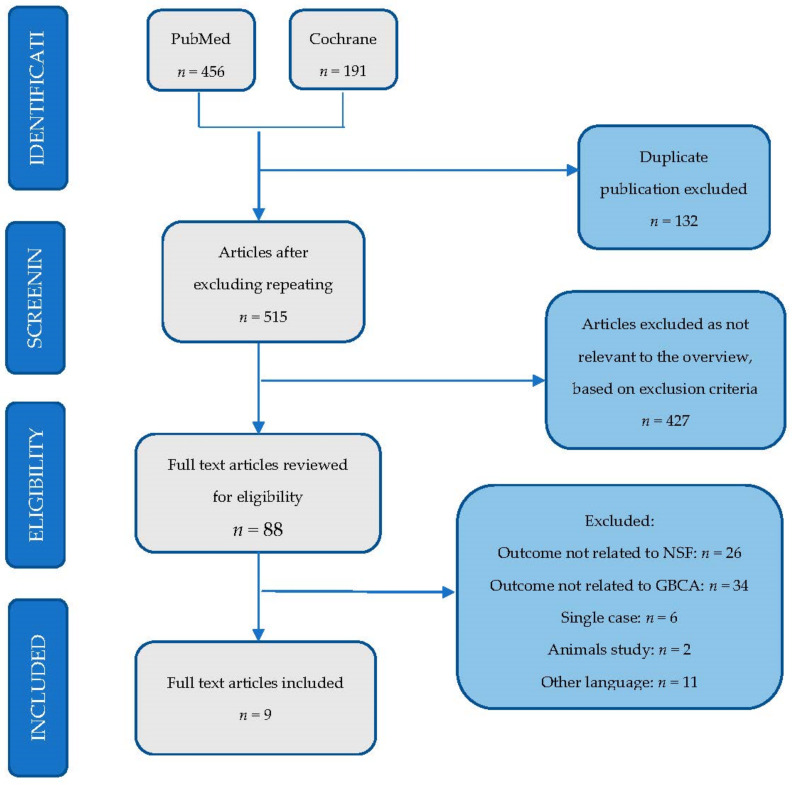
Scheme for articles that qualified for a systematic review.

The review included studies in which a gadolinium-based contrast agent was used during MRI in patients with kidney disease. A total of 647 articles were found in scientific databases. After the elimination of the duplicate articles, 515 papers remained for analysis. In the next phase, after reviewing the summaries, 88 full-text articles were preserved. The last stage focused on the inclusion and exclusion criteria. Finally, nine articles were accepted for systematic analysis (Figure 1).

A total of 173 cases with NSF were included in the analysis (Table 5). The majority of patients were undergoing dialysis. The contrast medium used for MRI was most often Omnican and Magnevist. The dose of contrast agent used varied from study to study. Due to the use of unequal units, it is not possible to calculate an average dose of contrast agent used. The shortest time for symptoms of NSF was two days. The diagnosis of NSF was also made three years after the use of a contrast agent. The authors of two studies did not determine the time of occurrence of NSF symptoms from exposure to contrast medium. Three authors pointed out in their papers other factors that could potentially influence the occurrence of NSF. These included: metabolic acidosis, ongoing infection, higher doses of erythropoiesis-stimulating agents and higher serum concentrations of ionized calcium and phosphate.

### 3.1. Demographic and Social Data

Reports of the NSF case came from four countries (Austria, Denmark, USA, Germany). The distribution for the 161 cases for which gender was available was as follows: females, 74 (46%) cases; males, 87 (54%) cases. For patients with the given age, the average age was 55 years. The above data are presented in Table 6.

### 3.2. Dialysis

Of the cases described, 130 (75.1%) patients were on dialysis. Conservative treatment was provided to 38 patients (22%). For five cases (2.9%), no data on treatment were obtained. The authors of seven articles (83 cases) divided patients according to the type of dialysis performed. Among these patients, the vast majority underwent hemodialysis. They constituted a group of 71 (85.5%) people. Peritoneal dialysis was over 12 (14.5%), as shown in Table 7.

### 3.3. Contrast Agents, Duration of NSF Symptoms

Most of the described cases were exposed to linear gadolinium chelates during magnetic resonance imaging. These were gadodiamide (Omniscan) and gadopentetate dimeglumine (Magnevist). Only in a few cases were contrasting mediums of cyclic structure used. The average amount of contrast used in the study is impossible to determine due to the discrepancy in the units determining the amount of given contrast. However, it can be seen that some patients received a dose higher than the standard 0.1 mmol/kg. The time of exposure to NSF symptoms was different. In some cases, the first changes were observed as early as two days after exposure to a contrast agent, and in some cases, the diagnosis of NSF was made after three years. The above data are presented in Table 1.

### 3.4. Other Potential Risk Factors of NSF

The authors of five studies have pointed out other variables that occurred in patients with NSF, which may also be a factor increasing the development of the disease. In one study, all patients who developed NSF had metabolic acidosis, while healthy patients showed normal pH values [5]. Others noted elevated inflammatory values during contrast administration in patients who developed NSF [22,24]. Marckmann et al., suggest that the risk of NSF increases during therapy with higher doses of erythropoietin and higher serum concentrations of calcium and phosphorus in patients at the time of exposure to contrast agents [8].

## 4. Discussion

NFS is undoubtedly a serious complication that can lead to significant disability, invalidity and even death. The Tood et al. study analyzed the mortality of people who suffered from NSF. The mortality rate within 24 months was significantly higher in patients with NSF and was 48%, while in those with no NSF it was 20% [7].

The hypothesis that gadolinium contrast agents are related to the development of NSF was put forward for the first time by Grobner et al., in 2006 [5]. The common feature of all patients with NSF is renal failure. Many authors emphasized that the highest risk of NSF exists in patients with a GFR < 15 mL/min/1.73 m^2^ (i.e., chronic kidney disease at stage 5) [7,22,23,24] In patients with kidney failure, the period of the elimination of the contrast medium from the body can be extended up to over 30 h. The role of dialysis in preventing NSF is unclear. Currently, it is assumed that the risk of developing NSF may decrease when patients undergo immediate dialysis (<24 h) after contrast medium administration [25]. However, Saitoh et al., pointed out that one dialysis treatment is not sufficient to remove all gadolinium [13]. In a study by Perez-Rodrigue et al., seven patients underwent dialysis within 24 h of contrast medium exposure and continued to develop NSF [24]. Some authors suggest that it might be acceptable to give some additional dialysis sessions to patients already undergoing renal replacement therapy. It would, at least, limit the potential Gd3+ toxicities [29].

Metabolic acidosis, ongoing inflammatory process, treatment with erythropoietin and high serum concentrations of calcium and phosphorus have been reported as factors contributing to the development of NSF [5,7,23,26,30,31]. In the Grobner et al. study, all patients affected by NSF had metabolic acidosis. Their mean pH was 7.29 ± 0.04 (mean actual bicarbonate value 19.5 ± 1.7 mmol/L), while the mean pH of healthy individuals was 7.39 ± 0.01 (mean actual bicarbonate value: 22.95 ± 0.58 mmol/L) [5]. Marckmann et al., did not confirm this hypothesis. In their clinical–control study, no significant difference in serum bicarbonate concentration between patients with NSF and the control group was found. However, the researchers pointed out the difference in treatment with erythropoietin. Ultimately, the hypothesis that NSF may be induced by analogs of erythropoietin was rejected because three of their subjects have never been treated with such drugs. However, there was a tendency to use higher doses of erythropoietin in people with NSF than in the control group. Higher doses of erythropoietin were used in people with a severe course of NSF [8]. Of note, a series of cases described by Othersen et al., did not support this hypothesis—two patients received a small dose of erythropoietin, and two did not take it while exposed to gadolinium [27]. The researchers suggest that in the case of erythropoietin treatment, the relationship with NSF is unlikely to be causative [12,27,32]. In a study by Marckmann et al., it was noted that all NSF cases had significantly higher concentrations of phosphate and ionized calcium in serum during exposure to contrast media. This confirms the chemical theory that higher levels of ionized calcium and phosphates lead to a process of transmetallation and a greater risk of Gd3+ ion retention outside the contrast agent complex. This leads to the retention of Gd3+ ion, which is toxic to the body, and its penetration through membranes to other cells [8,22]. Marckmann et al., in their study, found a borderline higher incidence of calcium supplements in patients with a severe course of NSF compared to mild cases [8].

A significant relationship between NSF and infection was confirmed by Golding et al. Precisely 6.7% of NSF cases were with an infection. It has been estimated that infection in patients with renal failure increases the risk of NSF 25-fold [23]. Becker et al., point out that although in their series of cases a large group of patients also presented signs of inflammation, it is difficult to estimate their contribution to the development of NSF. Elevated inflammatory markers are often present in patients with chronic kidney disease [26]. Moreover, some contrast agents themselves may cause acute inflammation [32,33].

In 2007, the US Food and Drug Administration (FDA) mandated a black box warning advising the avoidance of all gadolinium-containing contrast agents in at-risk patients. The label was updated in 2010. It included a recommendation to perform the screening of renal function tests, reduce the dose of contrast and use lower-risk contrasts. Implementation of the recommendations and changes in hospital policy have contributed to virtually eliminating this complication [34,35]. Since 2008, the number of reported NSF cases has decreased significantly. This may show the adherence to regulatory recommendations to avoid GBCAs in patients with a GFR less than 30 mL/min/1.73 m^2^ [36]. More recent guidelines and reports show that not all contrast agents have the same risk of triggering NFS [37]. The lowest risk contrast agents are: gadobenate dimeglumine, gadoteridol, gadoterate meglumine and gadobutrol [38]. A systematic review and meta-analysis risk of NFS in Patients with Stage 4 or 5 Chronic Kidney Disease Receiving a Group II Gadolinium-Based Contrast Agent by Woolen et al., showed that the use of contrasts with the lowest risk in patients with kidney disease in stage 4 and 5 CKD is less than 0.07% [39]. The risk of developing NSF is almost as small as the risk of developing an allergic reaction after contrast application, with frequencies varying from 0.004% to 0.7% [38].

NSF is not an entity of the past, and the FDA continues to receive reports on new cases of NFS each year through the US Food and Drug Administration Adverse Reporting System Public Dashboard. There were 32 cases each reported in 2019 and 2020 [35].

## 5. A Limitation of the Systematic Review

One limitation of our review was the small pool of confirmed NSF cases. We focused only on patients with renal failure who had gadolinium-based contrast administered during the MRI scan. The systematic review is limited to studies conducted between 2006 and 2020, and after 2008, the number of reported cases of NSF related to gadolinium contrast agent exposure decreased significantly (only seven biopsy-confirmed patients). The results of the review concerned only full-text papers that we were able to get through the library of our university.

## 6. Conclusions

GBCAs have been in clinical use for more than 30 years. NFS remains a rare but serious complication of their application. Gadolinium-induced systemic fibrosis leads to disability and eventually even death. After reviewing the available studies, we found that most of the described cases of NSF occurred in middle-aged people with acute or chronic kidney disease. Most NSF occurs after exposure to linear contrast agents. However, this should not result in a delay or the complete refusal of contrast-enhanced MRI in patients with renal disease. The risk of developing NSF after gadolinium-containing contrast agents is variable. Recent reports indicate that the use of group II contrast agents carries a low risk of complications in patients with renal disease. There are numerous recommendations made by professional societies regarding the use of GBCA in patients at risk for NSF [29]. In patients with a GFR < 30 mL/min, contrast agents with the lowest risk of developing NSF are recommended. The implementation of the new rules and increased awareness of the risk of complications among radiology staff has resulted in a spectacular decrease in the incidence of NSF in recent years.

Nevertheless, vigilance needs to be maintained, and further studies and observations on the incidence of this serious complication should be conducted.

## Figures and Tables

**Table 1:** Characteristics of contrast agents.

Trade Name	Generic Name	Chemical Structure	Charge	Elimination Way	Risk of NSF *	ACR Classification of GBCA **
Omniscan ^®^	Gadodiamide	Linear	Nonionic	Kidney	High	Group I
OptiMARK ^®^	Gadoversetamide	Linear	Nonionic	Kidney	High	Group I
Magnevist ^®^	Gadopentetate dimeglumine	Linear	Ionic	Kidney	High	Group I
MultiHance ^®^	Gadobenate dimeglumine	Linear	Ionic	97% Kidney 3% Bile	Medium	Group II
Primovist ^®^	Gadoxetate disodium	Linear	Ionic	50% Kidney 50% Bile	Medium	Group III
Dotarem ^®^	Gadoterate meglumine	Cyclic	Ionic	Kidney	Low	Group II
ProHance ^®^	Gadoteridol	Cyclic	Nonionic	Kidney	Low	Group II
Gadovist ^®^	Gadobutrol	Cyclic	Nonionic	Kidney	Low	Group II

* According to the Committee for Medicinal Products for Human Use (CHMP). ** American College of Radiology Classification (ACR) of gadolinium-based contrast agents relative to risk of nephrogenic systemic fibrosis (NFS).

**Table 2:** Terms used in the search.

Databases Used	PubMed ^1^			Cochrane Library ^1^		
Strategies Used	Indexed Search Terms	Free Text Words	Combination of Free Text Words and Indexed Terms	Indexed Search Terms	Free Text Words	Combination of Free Text Words and Indexed Terms
Participant/Patient ^2^	“Renal failure” (MeSH)	Renal failure	Renal failure	MeSH descriptor: (renal failure)	Renal failure	MeSH descriptor: (renal failure) * Renal failure
Intervention ^3^	“Gadolinium” (MeSH)	Contrast medium, *MRI	Gadolinium Contrast medium, MRI	MeSH descriptor: (gadolinium)	Contrast medium, MRI	MeSH descriptor: (gadolinium) Contrast medium, * MRI
Comparison ^4^	“nephrogenic systemic fibrosis” (MeSH)	NSF	Nephrogenic systemic fibrosis *NSF	MeSH descriptor: (nephrogenic systemic fibrosis)	NSF	MeSH descriptor: (nephrogenic systemic fibrosis) NSF
Number of Systematic Review Retrieved	3	36	39	1	21	26
Articles Chosen after Title Screening	2	12	15	1	3	6
Articles Chosen after Abstract Screening	1	3	6	1	2	3

^1^ Cochrane and PubMed both shared index terms from the same source, which is also known as the Medical Subject Headings (MeSH) thesaurus. ^2,3,4^ All search terms in one element are linked with the “OR” Boolean operator, after which search terms between different elements are linked by the “AND” Boolean operator.

**Table 3:** Quality assessment of the included studies by the Newcastle–Ottawa Scale.

First Author, Year	Study Design	Selection	Comparability	Exposure/Outcome	Total Scores
Deo A. et al., 2007 [3]	Retrospective cohort study	***	*	***	7
Grobner T et al., 2006 [5]	Observational cohort study	***	**	**	7
Marckmann P. et al., 2006 [8]	Retrospective cohort study	***	*	***	7
Marckmann P. et al., 2007 [22]	Case–control studies	***	**	***	8
Golding L.P. et al., 2007 [23]	Observational cohort study	***	**	**	7
Perez-Rodrigue J. et al., 2009 [24]	Retrospective cohort study	**	*	***	6
Elmholdt T. et al., 2013 [25]	Retrospective cohort study	***	**	**	7
Becker S. et al., 2010 [26]	Retrospective cohort study	***	**	**	7
Othersen J. et al., 2007 [27]	Observational cohort study	***	*	***	7

* A study can be awarded a maximum of one star for each numbered item within the Selection and outcome categories (categories selection max.4 stars; categories Comparability max.1 star; categories Exposure/Outcome max.3 stars).

**Table 4:** Summary of the AMSTAR 2 quality assessment.

	Deo A. et al., 2007 [3]	Grobner T et al., 2006 [5]	Marckmann P. et al., 2006 [8]	Marckmann P. et al., 2007 [22]	Golding LP. et al., 2007 [23]	Perez-Rodrigue J. et al., 2009 [24]	Elmholdt T. et al., 2013 [25]	Becker S., et al., 2010 [26]	Othersen J. et al., 2007 [27]	Total, N (%)
(1) Question and Inclusion	Yes	Yes	Yes	Yes	Yes	Yes	Yes	Yes	Yes	9(100%)
(2) Protocol	No	No	No	No	No	No	No	No	No	0(0%)
(3) Study Design	Yes	Yes	Yes	Yes	Yes	Yes	Yes	Yes	Yes	9(100%)
(4) Comprehensive Search	Yes	Yes	Yes	Yes	Yes	Yes	Yes	Yes	Yes	9(100%)
(5) Study Selection	Yes	Yes	Yes	Yes	Yes	Yes	Yes	Yes	Yes	9(100%)
(6) Data Extraction	Yes	Yes	Yes	Yes	Yes	Yes	Yes	Yes	Yes	9(100%)
(7) Excluded Studies Justification	Yes	Yes	Yes	Yes	Yes	Yes	Yes	Yes	Yes	9(100%)
(8) Included Studies Details	Yes	Yes	Yes	Yes	Yes	Yes	Yes	Yes	Yes	9(100%)
(9) Risk of Bias (RoB)	No	No	No	No	No	No	No	No	No	0(0%)
(10) Funding Sources	No	No	No	No	No	No	No	No	No	0(0%)
(11) Statistical Methods	Yes	No	Yes	Yes	Yes	Yes	No	No	Yes	6(67%)
(12) RoB on Meta-Analysis	No	No	No	No	Yes	No	No	No	No	1(11%)
(13) RoB in Individual Studies	Yes	Yes	Yes	Yes	Yes	Yes	Yes	Yes	Yes	9(100%)
(14) Explanation for Heterogeneity	Yes	Yes	Yes	Yes	Yes	Yes	Yes	Yes	Yes	9(100%)
(15) Publication Bias	Yes	Yes	Yes	Yes	Yes	Yes	Yes	Yes	Yes	9(100%)
(16) Conflict of Interest	Yes	Yes	Yes	Yes	Yes	Yes	Yes	Yes	Yes	9(100%)

Abbreviations: RoB, risk of bias. Percent is based on the number of eligible reviews per domain.

**Table 5:** Analysis of articles included in the review.

First Author, Year	Number of NSF Cases	Average Age	Treatment of Kidney Disease	Contrast Agent	Amount of Contrast Agent	Time of Occurrence of NSF Symptoms from Exposure (Days)	Other Potential Factors
Deo A. et al., 2007 [3]	3	60	3, dialysis	Omniscan Magnevist	20–125 mL	60	-
Grobner T et al., 2006 [5]	5	57	5, dialysis	Magnevist	16.3–20.7 mmol/L	14–28	Metabolic acidosis
Marckmann P. et al., 2006 [8]	13	50	8, dialysis 5, no dialysis	Omniscan	9–25 mmol/L	2–75	-
Marckmann P. et al., 2007 [22]	19	52	7, dialysis 7, no dialysis	Omniscan	0.18–0.50 mmol/kg	No data	Higher doses of erythropoietin; higher serum concentrations of ionized calcium and phosphate
Golding LP. et al., 2007 [23]	7	56	6, dialysis 1, no dialysis	Omniscan	0.10–0.32 mmol/kg	2–150	Infection
Perez-Rodrigue J. et al., 2009 [24]	33	49	25, dialysis 8, no dialysis	Omniscan Magnevist	12–80 mL	14–112	-
Elmholdt T. et al., 2013 [25]	65	53	44, dialysis 16, no dialysis 5, no data	Omniscan Magnevist Dotarem Gadovist Multihance	31.5 mL	No data	-
Becker S. et al., 2010 [26]	23	61	21, dialysis 2, dialysis	Omniscan Magnevist Gadovist	No data	1 days–3 years	Infection
Othersen J. et al., 2007 [27]	5	No data	5, dialysis	Omniscan Magnevist Multihance	7.5–10 mmol	60–90	-

**Table 6:** Demographic and social data.

First Author, Year	Country	Gender		
		Females	Males	No Description
Deo A. et al., 2007 [3]	USA	1	2	-
Grobner T. et al., 2006 [5]	Austria	3	2	-
Marckmann P. et al., 2006 [8]	Denmark	8	5	-
Marckmann P. et al., 2007 [22]	Denmark	10	9	-
Golding L.P. et al., 2007 [23]	USA	-	-	7
Perez-Rodrigue J. et al., 2009 [24]	USA	13	20	-
Elmholdt T. et al., 2013 [25]	Denmark	28	37	-
Becker S. et al., 2010 [26]	Germany	11	12	-
Othersen J. et al., 2007 [27]	USA	-	-	5
Total		74	87	12
		173		

**Table 7:** Treatment of kidney disease.

First Author, Year	Number of NSF Cases	Type of Dialysis			Conservative Treatment	Not Recorded
		Hemodialysis	Peritoneal Dialysis	Not Described		
Deo A. et al., 2007 [3]	3	-	-	3	0	-
Grobner T. et al., 2006 [5]	5	5	0	-	0	-
Marckmann P. et al., 2006 [8]	13	7	1	-	5	-
Marckmann P. et.al., 2007 [22]	19	9	3	-	7	-
Golding L.P. et.al., 2007 [23]	7	6	0	-	1	-
Perez-Rodrigue J. et al., 2009 [24]	33	20	5	-	8	-
Elmholdt T. et al., 2013 [25]	65	-	-	44	16	5
Becker S. et al., 2010 [26]	23	21	1	-	1	-
Othersen J. et al., 2007 [27]	5	3	2	-	0	-
Total		71	12	47	38	5
		173				

## Data Availability

The authors declare that the data of this research is available from the corresponding author on request.
